# Green-Chemical Strategies for Production of Tailor-Made Chitooligosaccharides with Enhanced Biological Activities

**DOI:** 10.3390/molecules28186591

**Published:** 2023-09-13

**Authors:** Reeba Thomas, Tamo Fukamizo, Wipa Suginta

**Affiliations:** 1 School of Biomolecular Science and Engineering (BSE), Vidyasirimedhi Institute of Science and Technology (VISTEC), Payunai, Wangchan District, Rayong 21210, Thailand; reeba.t_s19@vistec.ac.th (R.T.); tamo0111fuka@gmail.com (T.F.); 2 Department of Advanced Bioscience, Kindai University, 3327-204 Nakamachi, Nara 631-8505, Japan

**Keywords:** chitooligosaccharides, biological activities, chitinase, chitosanase, transglycosylation, glycosynthase

## Abstract

Chitooligosaccharides (COSs) are b-1,4-linked homo-oligosaccharides of *N*-acetylglucosamine (GlcNAc) or glucosamine (GlcN), and also include hetero-oligosaccharides composed of GlcNAc and GlcN. These sugars are of practical importance because of their various biological activities, such as antimicrobial, anti-inflammatory, antioxidant and antitumor activities, as well as triggering the innate immunity in plants. The reported data on bioactivities of COSs used to contain some uncertainties or contradictions, because the experiments were conducted with poorly characterized COS mixtures. Recently, COSs have been satisfactorily characterized with respect to their structures, especially the degree of polymerization (DP) and degree of *N*-acetylation (DA); thus, the structure–bioactivity relationship of COSs has become more unambiguous. To date, various green-chemical strategies involving enzymatic synthesis of COSs with designed sequences and desired biological activities have been developed. The enzymatic strategies could involve transglycosylation or glycosynthase reactions using reducing end-activated sugars as the donor substrates and chitinase/chitosanase and their mutants as the biocatalysts. Site-specific chitin deacetylases were also proposed to be applicable for this purpose. Furthermore, to improve the yields of the COS products, metabolic engineering techniques could be applied. The above-mentioned approaches will provide the opportunity to produce tailor-made COSs, leading to the enhanced utilization of chitin biomass.

## 1. Introduction

**Figure 1:**
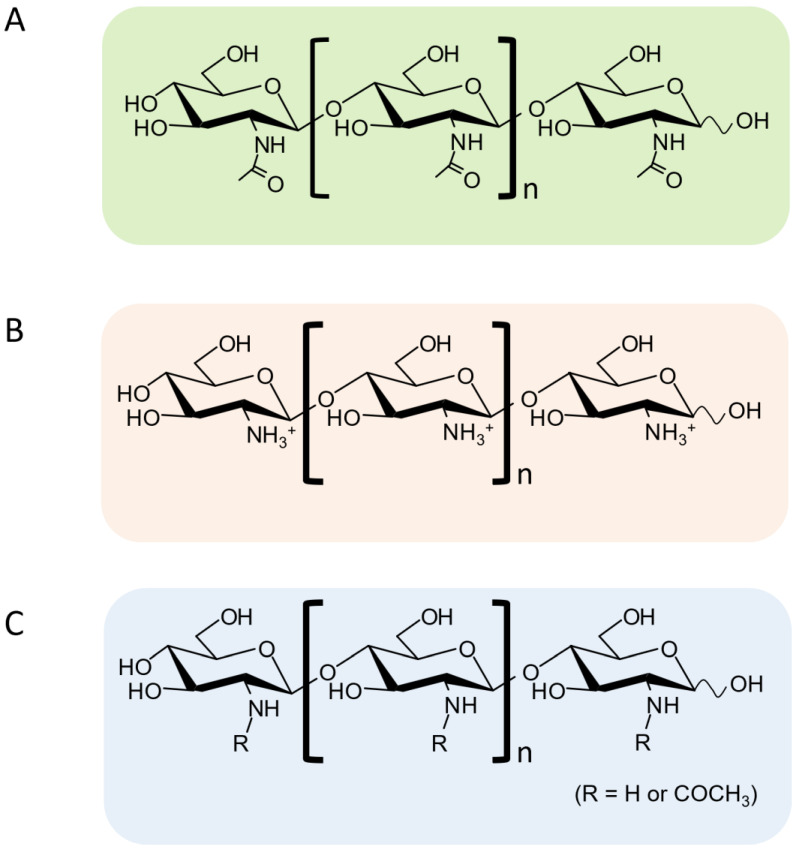
The molecular structures of COSs with different bioactivities. (**A**) Chitooligosaccharides (GlcNAc)_n_, n = 1, 2, 3, …, (**B**) chitosan oligosaccharides (GlcN)_n_, n = 1, 2, 3, …, (**C**) partially *N*-acetylated chitooligosaccharides, ······GlcNAc-GlcN······ n = 1, 2, 3, ….

Chitin, a b-1,4-linked polysaccharide of *N*-acetylglucosamine (GlcNAc), is the second most abundant biomass on the earth, next to cellulose. Three forms of chitin are present; α-chitin, the most abundant and tightly compacted crystalline structure, is arranged in an anti-parallel fashion [1,2]. α-chitin forms are commonly found in arthropods including crabs, lobsters, and shrimp, and also in fungal cell walls [3]. β-chitin, in contrast to α-chitin, has weak hydrogen bonding arranged in parallel forms, resulting in weak intramolecular forces, and is distributed in diatoms, squid pens, and annelids [4]. The least form is γ-chitin, which contains a mixture of both parallel and anti-parallel forms [4]. De-*N*-acetylated derivatives of chitin, chitosan, are mainly found in fungal cell walls and exhibit two forms, Type I and Type II [5,6]. Modern solid-state NMR revealed the polymorphism of these polysaccharide structures [7,8]. Although a similar polysaccharide, cellulose (a glucan having b-1,4-glycosidic linkages), is most efficiently utilized as an essential material in our daily life, the utilization of chitin and chitosan is much less intensive. Many attempts have been made to enhance the utilization of these polysaccharides [9], but effective approaches still remain to be developed. In previous attempts at enhancing chitin utilization, chitin and chitosan were degraded to chitooligosaccharides (COS) by acid or enzymatic hydrolysis [10], and the oligosaccharides were characterized with respect to their antimicrobial, anti-inflammatory, antioxidant, immune-potentiating, and antitumor activities [11]. Nowadays, COSs are well known to exhibit such biological activities in vitro or in vivo. These activity data were obtained using COS mixtures, which included b-1,4-linked oligosaccharides, composed of *N*-acetylglucosamine (GlcNAc), glucosamine (GlcN), or both (Figure 1); hence, they are not fully characterized with respect to their chemical structures.

**Figure 2:**
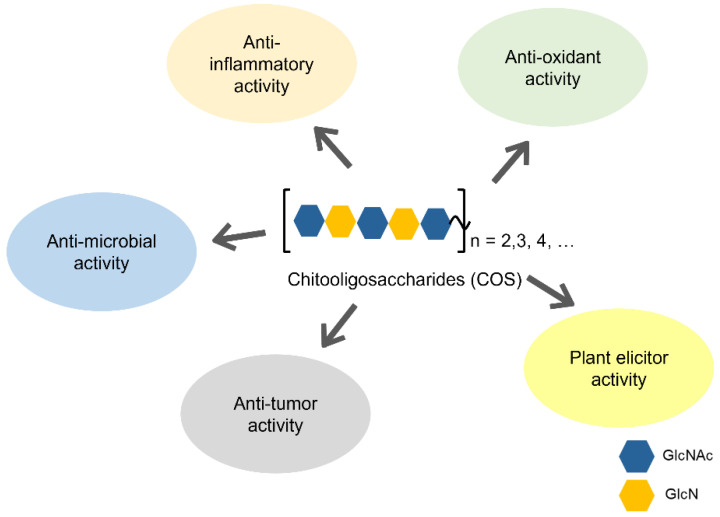
A summary of biological activity of COSs.

However, recent developments in separation and analytical methods for carbohydrates have enabled exact determinations of the degrees of polymerization (DP) and of *N*-acetylation (DA), as well as sequences, which can be analyzed by NMR, IR, and mass spectrometry [12,13,14,15]. In fact, the biological activities of COS were found to be controlled by the three structural factors: DP, DA, and sequence. For example, the antioxidant and anti-inflammatory activities of COS were dependent on their DP, as well as on DA [16,17]. Similar dependencies were also observed for other biological activities, as described previously [11]. Furthermore, it was proposed that the specific sequences composed of GlcNAc and GlcN, so called “ChitoCode”, were recognized by some proteins with significant functions in fungal interactions with other organisms [18]. Similar mechanisms of molecular recognition may exist in other interaction systems composed of living organisms that have chitin/chitosan as structural components. To obtain COSs with the desired biological activities, it has now become possible to design and synthesize COSs with specified sequences, so called tailor-made COSs. Here, we have made a comprehensive review of the biological activities of COSs and discussed their structure–function relationship. This review also underlines the green-chemical methods reported for the synthesis of pure COSs with designated sequences. These methods are considered promising approaches for the production of high-quality COSs with enhanced biological activities (Figure 2).

## 2. Biological Activities of COSs

### 2.1. Antimicrobial Activity

COSs have been recognized to exhibit antimicrobial activities, inhibiting the growth of a variety of phytopathogenic and animal-pathogenic fungi, bacteria, and viruses. The bactericidal activities of COSs with higher (5–10 kDa), medium (1–5 kDa), and lower molecular weights (<1 kDa) were evaluated by counting the colony formed on the medium, comparing to the control, and found to be 98, 62, and 51% toward *Escherichia coli* (Gram-negative); 47, 35, and 22% toward *Pseudomonas aeruginosa* (Gram-negative); 82, 57, and 23% toward *Staphylococcus epidermidis* (Gram-positive); and 98, 63, and 57% toward *Lactobacillus bulgaricus*, respectively [19]. COSs with higher molecular weights had greater antimicrobial effects than those with medium and lower ones. Higher DPs were also reported to be beneficial for exerting the antibacterial activities of COSs [20,21]. It is likely that the positive charges of COSs interact with negative charges on the bacterial cell surfaces, leading to inhibition of cell growth. In contrast, the shorter COSs (DA = 0.15; DP = 2, 3, and 4) and low molecular weight chitosan produced by a chitosanase from *Bacillus thuringiensis* B-387 from chitosan polysaccharide (DA, 0.15), of which the average MWs were in a range of 14–46 kDa, exhibited greater antibacterial activities toward *E. coli* in vitro (MIC, 70 mg·mL^−1^) in comparison with the initial polysaccharides (MIC, 120 mg·mL^−1^) [22]. Ganan et al. analyzed the antifungal activities of soluble and well-defined COSs. Determination of the antifungal activities of COS fractions with varying average DPs (17, 31, 54, and 62) indicated that fractions with intermediate DPs (31 and 54) had the strongest inhibitory effects; MICs toward *Candida guillermondii* were 78, 39, 39, and 1250 mg·mL^−1^, respectively [23]. Another report proposed that short-chain COSs exhibited high fungicidal activities toward the *Candida* species [24]. The contradictions in these findings may arise from differences in COS-preparation methods and the targeted microorganisms, as well as in experimental conditions. The antimicrobial activities may depend not only on the electrostatic interactions of free amino groups of COSs but also on the interaction of GlcNAc residues with the hydrophobic regions of microbial cell surfaces. A summary of the antimicrobial activities of COSs is presented in Table 1.

### 2.2. Anti-Inflammatory Activity

Four COSs with different DAs (0, 0.12, 0.5, and 0.85) and the same DP distributions were employed for assays of anti-inflammatory activity [17]. The results revealed that COSs with a DA of 0.12 had greater anti-inflammatory activity than COSs with other DAs. Another study demonstrated that low-MW COSs with DP of 3–5 obtained from chitosan were found to be effective against allergic inflammation in vivo [25]. In support of the fact that low-MW COSs possess the highest anti-inflammatory activity, Pangestuti et al. reported that depolymerized products from chitosan (MW < 10 kDa) reduced the production of nitric oxide and prostaglandin E2 by inhibiting inducible nitric oxide synthase (iNOS) and cyclooxygenase expression [26]. The effects were clearly dependent on the MW of COSs. The nitric oxide productions in BV2 microglia cell were about 10, 15, 20, and 23 mM for the treatment with 1, 1–3, 3–5, and 5–10 kDa COSs, respectively, while it was 25 mM in the control. The prostaglandin E2 productions were about 0.8, 1.0, 1.2, and 1.4 ng·ml^−1^, while it was 1.9 ng·ml^−1^ in the control. The effect of COSs (MW < 1 kDa) on nitric oxide production was further studied by Wei et al. [27]. Pretreatment with this COS could inhibit the production of nitric oxide by suppressing iNOS expression in activated microglial cells. Sanchez et al. [28] prepared COSs composed of 42% fully de-*N*-acetylated and 54% mono-*N*-acetylated oligomers through a single enzymatic hydrolysis step. This combination of COSs demonstrated anti-inflammatory effects [28]. Taken together, COSs with lower DPs (3–5) and a lower DA significantly attenuated the inflammation. It is notable, however, that completely de-*N*-acetylated COSs (DA = 0) exhibited no effective anti-inflammatory activity. The anti-inflammatory effects of COSs are summarized in Table 2.

### 2.3. Antioxidant Activity

Fully *N*-acetylated COSs (MW = 229.21–593.12 Da; DP = 1, 2, and 3) produced by the acid hydrolysis of crab chitin, which may contain a small fraction of de-*N*-acetylated residues, was found to inhibit myeloperoxidase activity and decrease free radicals in human myeloid cells, HL-60, or suppress oxidation of DNA and membrane proteins in mouse macrophages Raw 264.7 [29]. In the same work, COSs were shown to also directly scavenge free radical-induced DNA oxidation generated by 2′,7′-dichlorofluorescein, as well as increase intracellular glutathione levels, which confirms the antioxidant properties of COSs in living cells. Enzymatic hydrolysates from housefly larvae powder (HLP) contained mainly hetero-COS composed of GlcNAc and GlcN (DP = 2–6) [30]. Maximum production of COSs with a DP of 5 (51.2 μg·mL^−1^) was achieved by hydrolysis of HLP for 72 h and COSs with a DP of 5 exhibited the strongest hydroxyl-scavenging ability and reducing power. COSs obtained from chitosan with a DA of 0.05 have a protective effect on H_2_O_2_-induced human umbilical vein endothelial cell apoptosis and also from H_2_O_2_-induced oxidative damage in endothelial cells [31,32]. Five COSs from chitosan with increasing DPs (3–7) were examined and showed effects on the antioxidant activity of soybean (*Glycine max*) seeds during germination [33]. The COSs of different DPs exhibited varying influences on the antioxidant activity in soybean seeds. In particular, COSs with DP = 6 exerted a strong effect and significantly increased the antioxidant activity. Hao et al. [34] produced COS dimers with different sequences, GlcNAc-GlcNAc, GlcN-GlcNAc, GlcNAc-GlcN, and GlcN-GlcN, by the enzymatic method, and the antioxidant activities of all four dimers were studied. When 1 mg·ml^−1^ of the dimers was respectively added, the scavenging effects of the superoxide radicals were about 47 and 66% for GlcN-GlcN and GlcNAc-GlcN, respectively, and 12 and 8% for GlcN-GlcNAc and GlcNAc-GlcNAc. The amino group at the reducing end played a vital role in scavenging superoxide radicals. Furthermore, they found that GlcN-GlcN showed the highest 2,2-diphenyl-1-picrylhydrazyl scavenging activity. However, the scavenging effects of hydroxyl radicals were 45 and 42% for GlcNAc-GlcNAc and GlcN-GlcNAc, and 31 and 32% for GlcN-GlcN and GlcNAc-GlcN upon the addition of 4 mg·mL^−1^ of individual dimers. Although antioxidant activities were dependent on the radical species used in the experiments, GlcN residues in COSs were likely to dominate their antioxidant activity. COSs with a DP of 5–6 appeared to have higher antioxidant activity [30,33]. Antioxidant activities of COSs are summarized in Table 3.

### 2.4. Antitumor Activity

(GlcNAc)_6_ was found to display an anti-metastatic effect on tumor cells. Approximately 1 mg.kg^−1^ of (GlcNAc)_6_ administered intravenously resulted in a significant decrease in the pulmonary nodules formed from the Lewis lung carcinoma in mice [35]. However, the antitumor actions of COSs in vivo included various biological processes, such as absorption of COSs through the intestinal epithelium, distribution and accumulation in the kidney, liver, and spleen, enzymatic transformation, and elimination from the body. The DP, DA, and sequence of COSs may significantly affect the individual processes. Therefore, at present it is impossible to define clearly the structure–activity relationship for antitumor activity in vivo. Salah et al. [36] reported that low-MW chitin (2480 Da) obtained from shrimp shells was more effective against a human leukemia cell line than the higher-MW products (338 kDa chitin and 12 kDa chitosan). COSs with higher DA (0.15) exhibited 50 mg·mL^−1^ of CC_50_, the concentration of COSs required for 50% cell death of human PC3, A549, and HepG2 cells, while COSs with lower DA (0.01) exhibited 5–25 mg·mL^−1^ of CC_50_ [37]. A decrease in the MW or DA of COSs resulted in enhancement of the in vitro anticancer activity. However, it appeared that a DP of at least 6 is required to exert significant antitumor activity [11,38]. These findings on COS antitumor activities were recently reviewed by Zhai et al. [39]. The antitumor activities of COSs are outlined in Table 4.

### 2.5. Plant Elicitor Activity

Vander et al. [40] evaluated the capabilities of (GlcNAc)_n_ (n = 4, 5, 6, 7, 8, 9, and 10), (GlcN)_n_ (n = 5, 6, and 7) and partially *N*-acetylated chitosan to induce phenylalanine ammonia-lyase (PAL) and peroxidase (POD) in healthy wheat leaves. They found that purified (GlcN)_n_ were not active as plant elicitors, whereas purified (GlcNAc)_n_ (n ≥ 7) strongly elicited POD activities but not PAL activities. Partially *N*-acetylated chitosans elicited both PAL and POD activities, and maximum elicitation was observed with chitosan oligosaccharides with intermediate DAs. Ramakrishna et al. [41] reported the elicitor activities in rice seedlings of (GlcNAc)_n_ (n = 5–7) produced by a hypertransglycosylating mutant of a chitinase from *Serratia proteamaculans*. (GlcNAc)_7_ strongly induced an oxidative burst response, as well as peroxidase and PAL activities. These reports confirmed that (GlcNAc)_n_ (n = 5–7) are most effective in eliciting an immune response in plants. A chitin elicitor receptor kinase 1 (CERK1) was found to be essential for chitin perception by plant cells [42]. CERK1 was composed of an extracellular domain (ectodomain) with three contiguous LysM motifs (LysM1, LysM2, and LysM3), a transmembrane domain, and an intracellular kinase domain. Crystal structure analysis of the COS-bound ectodomain of CERK1 from *Arabidopsis thaliana* (*At*CERK1-ECD) [43] suggested that (GlcNAc)_8_ acted as a bivalent ligand that bound to the two *At*CERK1-ECD proteins through a continuous groove formed between one LysM2 and another LysM2, inducing the homo-dimerization of two *At*CERK1-ECD proteins. This dimerization formed an active receptor complex and was regarded as crucial for the immune response. In *A. thaliana*, (GlcNAc)_8_ binding to AtLYK5, another receptor containing three LysM motifs, induced the homo-dimerization, recruiting the two *At*CERK1 proteins and eventually forming an active receptor complex [44]. In rice, a chitin elicitor binding protein (*Os*CEBiP) composed of three LysM motifs [45] may first bind the (GlcNAc)_8_ elicitor, inducing the dimerization of *Os*CEBiP, and subsequently forming a hetero-tetramer receptor complex composed of two *Os*CEBiP and two *Os*CERK1. Hayafune et al. [46] found that hetero-COS DP-8 containing alternating GlcN and GlcNAc, (GlcN-GlcNAc)_4_, did not elicit an immune response in rice plants and hence was likely to inhibit formation of the hetero-tetramer complex. A hetero-COS with a DP of 6, GlcNAc-GlcN-(GlcNAc)_4_, elicited the immune response in *A. thaliana* but the response was in a lower level caused by (GlcNAc)_6_. In the case of another hetero-COS with a DP of 6, GlcNAc-GlcN-(GlcNAc)_2_-GlcN-GlcNAc, the immune response was eliminated almost completely [47]. Although the binding mode of COS elicitor to chitin receptor complex is still controversial [47], the *N*-acetylated residues were confirmed to be essential for the immune response in plants. The biological activities of COSs as plant elicitors are summarized in Table 5.

## 3. Synthetic Strategies of COSs with Promising Functions

Although the structure–function relationship of COSs is complex, the available data on COSs of various bioactivities provides information useful for the strategic design of tailor-made COSs with improved biological activities. To obtain COSs with desired sequences different green-chemical strategies have been reported, as described below.

### 3.1. Use of Transglycosylation (TG) Activity in Chitinolytic Enzymes

Many glycoside hydrolases with anomeric-retaining mechanism have been recognized to catalyze transglycosylation (TG) reactions to some extent [48]. Hen egg-white lysozyme is one of the enzymes that catalyze TG in addition to hydrolysis [49]. TG in the reaction catalyzed by lysozyme was estimated to be efficient from the HPLC-based reaction time-course of COS degradation [50,51,52,53] and was utilized for synthesis of COSs with longer chains and a higher degree of crystallinity [54]. Lysozyme TG was also applied to the synthesis of novel inhibitors for hen egg-white lysozyme [55] and a plant elicitor-active oligosaccharides [56]. Like lysozyme, chitinases belonging to the GH18 family were reported to catalyze TG reactions with COSs [57,58,59]. In these enzymatic TG reactions, native enzymes with full intrinsic hydrolytic activities were used. Therefore, COSs with longer chains produced by TG activity were again hydrolyzed to COSs with shorter chains. It was highly desirable to suppress the hydrolytic activity of these enzymes, although in this case complete loss of the glycosidic bond cleavage should be avoided, because the cleavage of the b-1,4-glycosidic bond activates the anomeric center to the transition state, which is essential for receiving the attack of the acceptor molecule [52,53]. It is necessary to employ some technique that suppresses hydration of the anomeric center in the transition state, while maintaining glycosidic bond cleavage activity. Fukamizo et al. reported enhanced TG activity in a chemically modified lysozyme, in which bulky residues, such as glucosamine and *p*-nitrophenyl-sulfenyl moieties, were introduced into the subsites −4, −3, and −2 of the lysozyme binding cleft [60]. They explained that the enhancement of TG activity was derived from the lower binding ability of the glycone-binding site (negatively numbered subsites), which, in turn, enhanced the acceptor binding to the aglycone-binding site (positively numbered subsites). However, the chemical methods seemed to be applicable only to inexpensive enzymes that are easy to isolate, such as lysozyme.

A GH2 exo-b-glucosaminidase from *Amycolatopsis orientalis* was reported to split off the non-reducing end GlcN unit from the (GlcN)_n_ (n = 2, 3, 4, …) substrates. This enzyme was found to catalyze a TG reaction in addition to hydrolysis; that is, from the (GlcN)_4_ substrate, (GlcN)_5_ and (GlcN)_6_ were formed as TG products [61]. After hydrolysis, the transition state of the GlcN monomer was transferred to the remaining (GlcN)_4_, which in turn acted as an acceptor, producing (GlcN)_5_. Similarly, the TG product (GlcN)_5_ subsequently acted as an acceptor, producing (GlcN)_6_. A GH5 chitosanase from *Streptomyces griseus* HUT 6037 was also reported to catalyze a TG reaction. When incubated with this enzyme, (GlcN)_5_ was hydrolyzed to (GlcN)_2_ + (GlcN)_3_ or (GlcNAc)_3_ + (GlcN)_2_. When the same reaction was conducted in the presence of an excess amount of (GlcNAc)_3_, TG products, including (GlcN)_2_-(GlcNAc)_3_ and (GlcN)_3_-(GlcNAc)_3_, were formed [62].

### 3.2. Mutation Strategies for Enhancing TG Activity

Various mutations have been attempted to enhance the TG activities of GH18 chitinases. Aronson et al. [63] reported for the first time that the mutation of a tryptophan residue in the substrate-binding cleft of *Serratia marcescens* chitinase A strongly enhanced the transglycosylation activity. The mutated tryptophan residue (Trp167) was located at subsite −3; therefore, the mechanism of TG enhancement was similar to that in the chemically modified lysozyme mentioned above [60]. Mutation of the middle Asp residue of the catalytic DxDxE motif of GH18 chitinases was also found to be useful for obtaining an efficiently transglycosylating chitinase [64,65]. Electrostatics in the catalytic cleft may be affected by the Asp mutation, probably resulting in the change in the state of the catalytic water molecule. Madhuprakash et al. [66,67] thoroughly mutated the amino acids localized in the catalytic center and the groove of a bacterial GH18 chitinase, indicating that the triple mutations, which reduce the hydrolytic activity, binding affinity, and stability of intermediate states, provided hypertransglycosylating mutants. Furthermore, they suggested the importance of optimal positioning of the catalytic water molecule and the acceptor molecule surrounding the catalytic center. In addition to these bacterial chitinases, plant chitinases were also employed in producing transglycosylating chitinases. Introducing the Trp side chain into the upper portion of the catalytic center of GH18 chitinases from *Arabidopsis thaliana* (*At*ChiC) and *Cycas revoluta* (*Cr*ChiA) was found to strongly enhance TG activities of the enzymes [68,69]. The enhanced hydrophobicity of this region may change the state of the catalytic water, resulting in the suppression of attack by the water molecule and thus enhancing the TG reaction. In particular, the *Cr*ChiA mutant, in which the Trp side chain was introduced, exhibited hypertransglycosylating activity [69].

### 3.3. Converting Chitinolytic Enzymes to Glycosynthase

**Figure 3:**
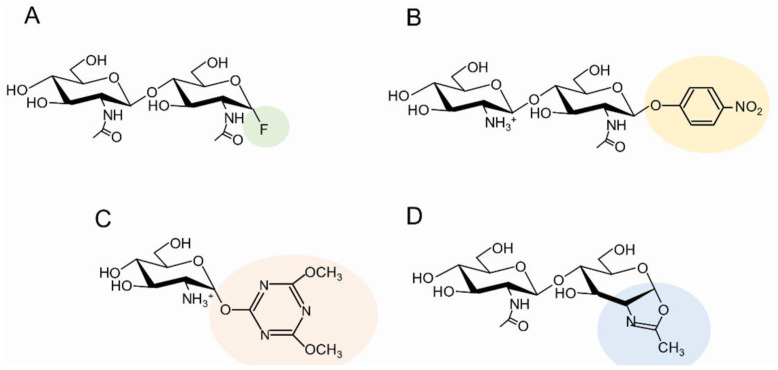
The chemical structures of activated COSs used for different green-chemical synthesis. (**A**) COS-fluoride, (**B**) *p*-nitrophenyl GlcN-GlcNAc dimer, (**C**) GlcN-DMP, (**D**) GlcNAc)_n_-oxazoline.

Glycosynthase was first developed from anomer-retaining glycoside hydrolases by mutation of the catalytic nucleophile [70,71]. In 2006, Honda and Kitaoka reported the first glycosynthase from an inverting glycoside hydrolase [72]. Since the retaining GH18 chitinases did not have a corresponding nucleophile in the catalytic center [73], inverting chitinases belonging to GH19 were employed for developing glycosynthase [74,75]. GH19 *Bryum coronatum* chitinase (*Bc*ChiA) mutants, in which the serine residue fixing a catalytic water molecule was mutated to alanine, cysteine, or glycine, successfully catalyzed glycosyl transfer of the activated COS fluoride (COS-F, Figure 3A) to the acceptor COS. Using these *Bc*ChiA mutants, (GlcNAc)_4_ was synthesized from (GlcNAc)_2_-fluoride (donor) and (GlcNAc)_2_ (acceptor). The substrate-binding groove of *Bc*ChiA consists of four subsites, −2, −1, +1, and +2, while that of a GH19 chitinase from rye seeds (RSC-c) consists of eight subsites, −4, −3, −2, −1, +1, +2, +3, and +4. It appeared that glycosynthase derived from RSC-c produces COSs with longer chains. In fact, the double mutants, in which Glu89, acting as a catalytic base and Ser120, fixing a catalytic water molecule, were mutated, produced (GlcNAc)_7_ from (GlcNAc)_3_-fluoride (donor) and (GlcNAc)_4_ (acceptor). Glycosynthase derived from hen egg-white lysozyme (HEL-D52S) was produced and found to catalyze the glycosyl transfer. Using GlcN-(GlcNAc)_2_-fluoride obtained from (GlcNAc)_3_-fluoride by the action of a site-specific chitooligosaccharide *N*-deacetylase, the HEL-D52S glycosynthase allowed the size-controlled synthesis of GlcN-(GlcNAc)_5_, GlcN-(GlcNAc)_6_, and GlcN-(GlcNAc)_7_ from the acceptors (GlcNAc)_3_, (GlcNAc)_4_, and (GlcNAc)_5_, respectively. The use of the site-specific deacetylase avoided a condensation reaction of the donor substrate itself [76].

### 3.4. Use of Activated Sugars as Donor Substrates

It is well known that *p*-nitrophenyl glycosides are efficient donor substrates in glycoside synthesis [77]. Harmsen et al. [78] reported the efficient synthesis of COSs with alternating GlcNAc and GlcN using *p*-nitrophenyl GlcN-GlcNAc dimer (GlcN-GlcNAc-*p*NP, Figure 3B), which was obtained by enzymatic deacetylation of GlcNAc-GlcNAc-*p*NP, and *Serratia* GH18 chitinase mutants as the catalysts. The mutants efficiently produced the alternating COSs composed of GlcN and GlcNAc, i.e., (DA)_2_, (DA)_3_, (DA)_4_, and (DA)_5_, which are possible candidates for inhibitors of chitin-related enzymes and proteins, such as human chitotriosidase, a therapeutic target for systemic sclerosis [78]. (DA)_4_ was also obtained from oxazoline derivatives of DA and was confirmed to be an inhibitor of chitin elicitor binding protein in plants [46].

Production of reducing end-activated COSs, such as glycosyl fluoride and *p*-nitrophenyl glycoside, used to require protection/deprotection procedures unfamiliar to enzymologists. Nowadays, however, protection-free methods for obtaining activated sugars have been developed and used for reducing end-activation of COSs. Tanaka et al. reported the protection-free synthesis of reducing end-activated sugar derivatives, in which the 4.6-dimethoxy-1,3,5-triazin-2-yl (DMT) group was introduced at the anomeric center of the reducing end [79]. GlcN-DMT (Figure 3C) was synthesized and used as the glycosyl donor for synthesizing (GlcN)_n_ with longer chains using GH2 exo-b-glucosaminidase as a template [80]. Furthermore, (GlcNAc)_2_-DMT (Figure 3C) was synthesized and used as a glycosyl donor for synthesizing (GlcNAc)_4_. A glycosynthase mutant derived from GH19 *Bc*ChiA comprising subsites −2, −1, +1, and +2 successfully synthesized (GlcNAc)_4_ from (GlcNAc)_2_-DMT and (GlcNAc)_2_ [81].

Sugar oxazolines have been frequently used as the glycosyl donors and are synthesized by the protection-free method using the water-soluble dehydrating agents, carbodiimides. Noguchi et al. reported that 2-chloro-1,3-dimethylimidazolinium chloride (DMC) was the most suitable for oxazoline synthesis from COSs. The yields were 70–80% [82]. The oxazoline derivatives from (GlcNAc)_n_ (Figure 3D; (GlcNAc)_n_-oxa, n = 2, 3, 4, and 5) were purified by HPLC and used as the donor substrates for synthesizing (GlcNAc)_n_ with longer chains using an activity-reduced mutant from bacterial GH18 chitinase as a template [83]. Various mutations were introduced into bacterial GH18 chitinases, *Sp*ChiD, and (GlcNAc)_10_ was produced from (GlcNAc)_5_-oxa in a good yield [84]. Hypertransglycosylating mutants of GH18 chitinases from plant origins, *Arabidopsis thaliana* and *Cycas revoluta*, were also used for (GlcNAc)_n_ synthesis from the sugar oxazolines [85]. The product distribution was controlled by using different combinations of the substrate size of donor/acceptor.

### 3.5. Use of Site-Specific Chitin Deacetylases

Hembach et al. [86] reported the enzymatic production of a full set of partially *N*-acetylated chitosan tetramers of all possible sequences consisting of GlcN and GlcNAc, through de-*N*-acetylation of (GlcNAc)_4_ using various site-specific chitin deacetylases and reverse *N*-acetylation of (GlcN)_4_ in the presence of acetate (2 M) by the same deacetylases. The structures of the products were successfully identified using ultra high-performance liquid chromatography–electrospray ionization–mass spectrometry (UHPLC/ESI/MS) [15,87], which enabled the full separation of the enzymatic products. In UHPLC/ESI/MS, a hydrophilic interaction chromatography (HILIC) column (Acquity UHPLC BEH Amide) was used to separate COS tetramers with different degrees of acetylation and sequences, and gradient elution was performed with 20–80% acetonitrile in water containing 10 mM NH_4_HCO_3_ and 0.1% (*v*/*v*) formic acid, at a flow rate of 0.8 mL.min^−1^ and 75 °C using an appropriate gradient program controlled by an UHPLC system purchased from Dionex Co. The effluents were detected with an ESI-MS detector in positive mode. The sequencing method of the COS tetramers consists of the following procedures: (1) acetylation of free amino groups in COSs using a deuterated reagent; (2) labeling the reducing end with H_2_^18^O; (3) quantifying COSs using [^13^C_2_, ^2^H_3_]-labeled internal standards; (4) sequencing by MS/MS [15]. The enzymatic de-*N*-acetylation/*N*-acetylation of the COSs, combined with the state-of-the-art separation/analysis methods, enabled the production of COSs with desired sequences. This may subsequently lead to defining the structure–function relationships of COSs. This strategy of using site-specific deacetylases was also utilized for producing chitosans with random and non-random acetylation patterns [88].

### 3.6. Metabolic Engineering Approaches

Studies of the biological activities of COSs usually need large amounts of COSs. However, the enzymatic methods described thus far provided only limited amounts of COSs. As an alternative to the current enzymatic methods, microbial fermentation using recombinant *Escherichia coli* has now attracted researchers’ attention, because the method is capable of producing larger amounts of COSs and is environmentally friendly [89]. Recently, a non-pathogenic bacterium, *Bacillus subtilis*, also provided an additional opportunity for the fermentative production of COSs [90]. *B. subtilis* was engineered by introducing a combinatorial pathway for the production of well-defined COSs. Specifically, an exogenous COS synthase was overexpressed in *B. subtilis*, then the GlcNAc synthesis module was also introduced to enhance the intracellular GlcNAc supply. Furthermore, both the de novo pathway and the salvage pathway of UDP-GlcNAc were engineered to further promote the biosynthesis of (GlcNAc)_n_. The metabolic engineering finally attained the production of (GlcNAc)_n_ of 4.8 g·L^−1^ including (GlcNAc)_5_ (86%), (GlcNAc)_4_ (7%), (GlcNAc)_3_ (5%), and (GlcNAc)_2_ (2%). Further engineering may provide a cell factory for producing the required amounts of COSs with desired DP, DA, and sequences [91].

## 4. Conclusions and Future Prospects

Recently, the relationship between the structures and the biological activities of COSs has become more explicit since the biological activities of COSs were satisfactorily characterized with respect to DPs, DAs, and sequences. Based on the data listed in Table 1, Table 2, Table 3, Table 4 and Table 5, it is possible to design COS sequences with desired biological functions. A combination of the strategies summarized in Table 6 enabled the synthesis of tailor-made COSs with specified sequences.

**Figure 4:**
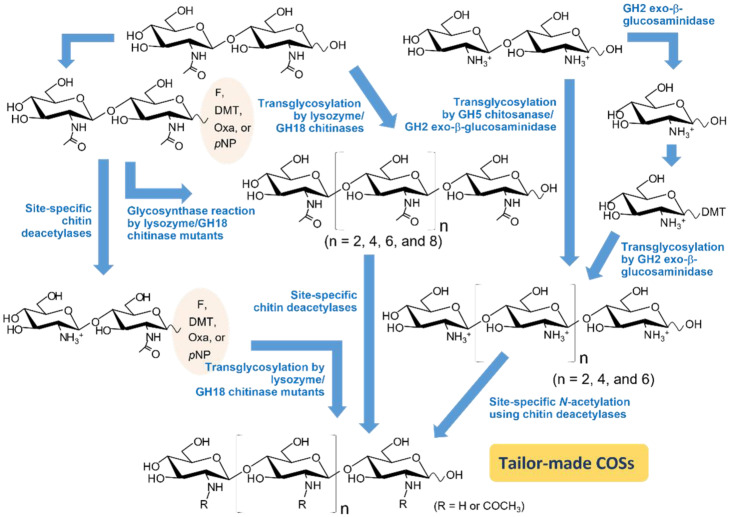
Proposed enzymatic synthesis of tailor-made COSs (DP4—10) through transglycosylation/glycosynthase reactions. Chitobiose and chitosan dimer are used as sugar acceptors and the reducing end-activated COSs are used as donor substrates. Site-specific de-*N*-acetylation/*N*-acetylation are subsequently used in the final step to generate tailor-made COSs.

It is well known that di-*N*-acetyl chitobiose (GlcNAc)_2_ is most efficiently produced from enzymatic degradation of chitin [92,93,94,95,96,97]. We successfully produced a large amount of high-quality (GlcNAc)_2_ from chitin food wastes using an in-house *Vibrio* chitinase [98], while (GlcN)_2_ was most efficiently produced by endo-splitting GH46 chitosanases from the chitosan wastes [99,100]. Starting from these chitobioses, various green-chemical strategies are proposed, as shown in Figure 4. COSs of differing chain length can be elongated from a DP of 2 to a DP of 4, 6, 8, or 10 by means of transglycosylation reactions, using chitinase/chitosanase mutants as effective catalysts. Glycosynthase mutants were also useful for chain-length elongation using reducing end-activated COSs as the donor substrates. Subsequent site-specific de-*N*-acetylation/*N*-acetylation may possibly produce tailor-made COSs that possess most plausible sequences with desired biological activities. The concept of tailor-made COSs will further enhance the exploitation of chitin biomass.

## Figures and Tables

**Table 1:** A summary of antimicrobial activity of COSs.

MW or DP	Source, DA, or Sequence	Activity	Assay	Ref.
Higher MW (5–10 kDa), Medium MW (1–5 kDa), and Lower MW (<1 kDa)	From chitosan	Higher MW > Medium MW > Lower MW	Growth inhibition toward Gram-positive, Gram-negative, and lactic acid bacteria	[19]
Chitosan polymers and Chitosan oligomers	From chitosan	Polymers > Oligomers	Growth inhibition toward *Staphyllococcus aureus* Gram-positive and Gram-negative bacteria	[20,21]
Chitosan polymers and DPs of 2–4, and Lower MW chitosans	Chitosan with DA, 0.15 or 0.5	DP of 2–4 and Lower MW chitosans > Initial polymers	Growth inhibition toward fungi and bacteria	[22]
Chitosans with average DPs of 17, 31, 54, and 62	From chitosan (DA, 0.15)	Chitosans with DP of 31 and 54 > Chitosans with DP of 17 and 62	Growth inhibition toward yeast, fungi, including *Candida*	[23]
Chitosans (70 and 600 kDa) Chitosan oligomers	From chitosan	Oligomers > Chitosans	Growth inhibition toward *Candida*	[24]

**Table 2:** A summary of anti-inflammatory bioactivity of COSs.

MW or DP	Source, DA, or Sequence	Activity	Assay	Ref.
COSs with similar DP distribution	Different DAs of 0, 0.12, 0.50, and 0.85	COSs with DA of 0.12 have the highest activity	Inhibition of LPS-induced inflammatory cytokine burst, down-regulating its mRNA expression, and reducing phosphorylation of IκBα	[17]
Chitosan oligomer mixture (DPs of 3–5)	DA of 0.0	Active toward allergic asthma inflammation in vivo	Inhibition of degranulation, cytokine generation in RBL-2H3 cells, and lung inflammation in allergic asthma model mice	[25]
Chitosan oligomers with MW < 1 kDa, 1–3 kDa, 3–5 kDa, and 5–10 kDa	From chitosan	The lower the MW, the higher the activity	Inhibition of iNOS and cyclooxygenase expressions	[26]
Chitosan oligomers with DP of 2–6 (weight percentages; 4, 16, 29, 37, and 14%, respectively)	From chitosan with DA < 0.05	Active toward N9 microglia cells	Inhibition of NO production by suppressing iNOS expression	[27]
42% fully de-*N-*acetylated/54% mono-*N-*acetylated oligomers (42/54) and 50% fully de-*N—*acetylated/27% mono-*N*-acetylated oligomers (50/27)	From chitosan	42/54 attenuated the inflammation both in vivo and in vitro, but 50/27 promoted the inflammatory response	Effects of COS preparations on inflammation in lipopolysaccharide-induced mice and in RAW264.7 macrophages	[28]

**Table 3:** A summary of antioxidant bioactivity of COSs.

MW or DP	Source, DA, or Sequence	Activity	Assay	Ref.
COS mixture with DP of 1, 2, and 3	Fully *N-*acetylated crab chitin (DA about 1.0)	Attenuated peroxidase activity Attenuated oxidation of DNA/membrane proteins Exhibited free-radical scavenging effects	Experiments using human myeloid cells, HL-60, and mouse macrophages RAW264.7	[29]
Hetero-COSs with DP of 2–6	From housefly larvae powder	COSs with major DP of 5 exhibited the highest activity	Hydroxyl-scavenging effects	[30]
Chitosan oligomers with DP of 2–6 (weight percentages: 4, 16, 29, 37, and 14%, respectively)	From chitosan with DA < 0.05	Protected from H_2_O_2_-induced apoptosis or oxidative damage	Experiments using human umbilical vein endothelial cell	[31,32]
COSs with different DPs (3–7)	From chitosan with DA of 0.0	COSs with DP of 6 had the highest activity	Effects on isoflavone contents and antioxidant activity in soyabean seeds during germination	[33]
COS dimers, (GlcNAc)_2_, GlcN-GlcNAc, GlcNAc-GlcN, and (GlcN)_2_	Site-specific chitin deacetylase treatments of (GlcNAc)_2_	(GlcN)2 > GlcNAc-GlcN >> GlcN-GlcNAc, (GlcNAc)2 toward superoxide radicals (GlcNAc)2 > GlcN-GlcNAc >> GlcNAc-GlcN, (GlcN)2 toward hydroxyl radicals (GlcN)2 >> GlcNAc-GlcN, GlcN-GlcNAc, (GlcNAc)2 toward DPPH	All scavenging effects were examined in vitro	[34]

**Table 4:** A summary of antitumor bioactivities of COSs.

MW or DP	Source, DA or Sequence	Activity	Assay	Ref.
(GlcNAc)_6_	From crab chitin	Decreased the pulmonary nodules	Experiments using Lewis lung carcinoma mice	[35]
Chitin, chitosan 2.5–338 kDa	From shrimp shell chitin	Chitin (2.5 kDa) > chitin (338 kDa), chitosan (12 kDa)	Experiments using human leukemia cells	[36]
Chitosan (DA, 0.015) and its enzymatic digestion products; COSs with DA, 0.0/DP, 3–5 and COSs with DA, 0.15/DP, 6–15	Enzymatic hydrolysis of high molecular weight chitosan with DA of 0.015	COSs with lower MW> COSs with higher MW COSs with lower DA> COSs with higher DA	Experiments using prostate and lung cancer cells, and hepatoma cells	[37]
Chitosan oligomers with DP of 2–6	Chemical and enzymatic hydrolysis of chitosan	DP should be at least 6 for antitumor action	Inhibitory effect on A549 cell proliferation	[38]

**Table 5:** A summary of COSs as plant elicitors.

MW or DP	Source, DA, or Sequence	Activity	Assay	Ref.
(GlcNAc)_n_ (n = 4, 5, 6, 7, 8, 9, and 10), (GlcN)_n_ (n = 5, 6, and 7) and partially *N-*acetylated chitosans	Chemical hydrolysis of chitin (fluorolysis)deacetylation of the high-MW chitin	(GlcN)n were not active as elicitors. (GlcNAc)n with a DP ≥7 elicited POD but not PAL. Partially N-acetylated chitosans elicited both PAL and POD.	Induction of phenylalanine ammonia-lyase (PAL), peroxidase (POD) in healthy wheat leaves	[40]
(GlcNAc)_n_ (n = 5, 6, and 7)	Enzymatic transglycosylation	(GlcNAc)_7_ induced oxidative burst as well as POD and PAL activities.	Induction of phenylalanine ammonia-lyase (PAL), peroxidase (POD), PR protein gene expression in rice seedlings	[41]
(GlcNAc)_8_ and hetero-COSs with a DP of 8	Enzymatic synthesis	(GlcNAc)_8_ was active, but (GlcN-GlcNAc)_4_ inactive.	Inhibition of CEBiP-dimerization and reactive oxygen generation	[46]
(GlcNAc)_6_ and two hetero-COSs with DP of 6	Enzymatic deacetylation of (GlcNAc)_6_	(GlcNAc)_6_ > GlcNAc-GlcN-(GlcNAc)_4_ > GlcNAc-GlcN-(GlcNAc)_2_-GlcN-GlcNAc.	Inhibition of reactive oxygen generation	[47]

**Table 6:** Strategies for synthesizing COSs with desired sequences.

Catalysts	Substrates		Products	Ref.
	Donor	Acceptor		
Hen egg-white lysozyme wild type	(GlcNAc)_3_		(GlcNAc)_n_ (n = 3–15)	[54]
Hen egg-white lysozyme wild type	(GlcNAc)_4_	Moranoline (1-deoxynojirimycin)	4-*O*-b-di(tri)-*N*-acetylchitobi(tri)osyl moranoline	[55,56]
Hen egg-white lysozyme Asp101, Trp62-modified	(GlcNAc)_5_		(GlcNAc)_9_	[60]
*Amycolatopsis orientalis* GH2 exo-b-D-glucosaminidase	(GlcN)_4_		(GlcN)_5_ and (GlcN)_6_	[61]
*Streptomyces griseus* HUT6037 GH5 endo-chitosanase	(GlcN)_5_	(GlcNAc)_3_	(GlcN)_2_-(GlcNAc)_3_ (GlcN)_3_-(GlcNAc)_3_	[62]
*Serratia marcescens* GH18 chitinase A mutated at Trp at site -3	(GlcNAc)_4_ or (GlcNAc)_5_		(GlcNAc)_6_ or (GlcNAc)_7_	[63]
*Serratia marcescens* GH18 chitinases A and B mutated at the middle Asp in the DxDxE motif	(GlcNAc)_4_		(GlcNAc)_3_ produced through the transglycosylation product (GlcNAc)_6_	[64]
*Vibrio harveyi* GH18 chitinase A mutated at the middle Asp in the DxDxE motif	(GlcNAc)_4_ (GlcNAc)_6_		(GlcNAc)_6_ (GlcNAc)_8_	[65]
*Serratia proteamaculans* GH18 chitinase D triple-mutated at the glycon- or aglycon-binding aromatic residues as well as at the middle Asp in the DxDxE motif	(GlcNAc)_4_		(GlcNAc)_5_ or (GlcNAc)_6_	[66]
*Serratia proteamaculans* GH18 chitinase D single-mutated at the catalytic center and the binding groove	(GlcNAc)_4_		(GlcNAc)_5_ or (GlcNAc)_6_	[67]
*Arabidopsis thaliana* GH18 chitinase C mutant, in which tryptophan side chain was introduced into the upper portion of the catalytic center	(GlcNAc)_4_		(GlcNAc)_3_ produced through the transglycosylation product (GlcNAc)_6_	[68]
*Cycas revoluta* GH18 chitinase A mutant, in which tryptophan side chain was introduced into the upper portion of the catalytic center	(GlcNAc)_4_		(GlcNAc)_3_ produced through the transglycosylation product (GlcNAc)_6_	[69]
A glycosynthase derived from *Bryum coronatum* GH19 chitinase A	(GlcNAc)_2_-fluoride	(GlcNAc)_2_	(GlcNAc)_4_	[74]
A glycosynthase derived from *Secale cereale* GH19 chitinase C	(GlcNAc)_3_-fluoride	(GlcNAc)_4_	(GlcNAc)_7_	[75]
A chitin-oligosaccharide *N*-deacetylase (NodB) and a glycosynthase derived from hen egg-white lysozyme (Asp52 → Ser)	GlcN-(GlcNAc)_2_-fluoride	(GlcNAc)_3_ (GlcNAc)_4_ (GlcNAc)_5_	GlcN-(GlcNAc)_5_ GlcN-(GlcNAc)_6_ GlcN-(GlcNAc)_7_	[76]
Hypertransglycosylating mutants from *Serratia marcescens* GH18 chitinases A and *Serratia proteamaculans* GH18 chitinase D	GlcN-GlcNAc-*p*NP (*p*-nitorophenylated) obtained by enzymatic de-*N*-acetylation of (GlcNAc)_2_-*p*NP		(GlcN-GlcNAc)_2_ (GlcN-GlcNAc)_3_ (GlcN-GlcNAc)_4_ (GlcN-GlcNAc)_5_	[78]
*Amycolatopsis orientalis* GH2 exo-b-glucosaminidase	GlcN-DMT (4,6-dimethoxy-1,3,5-triazin-2-yl)	(GlcNAc)_2_	GlcN-(GlcNAc)_2_	[80]
A glycosynthase derived from *Bryum coronatum* GH19 chitinase A	(GlcNAc)_2_-DMT	(GlcNAc)_2_	(GlcNAc)_4_	[81]
An activity-reduced mutant from *Bacillus circulans* GH18 chitinase A1	(GlcNAc)_2_-oxa	(GlcNAc)_5_	(GlcNAc)_7_	[83]
Catalytic-site mutants from *Serratia proteamaculans* GH18 chitinase D	(GlcNAc)_5_-oxa		(GlcNAc)_10_	[84]
Hypertransglycosylating mutants from *Nicotiana tobaccum* GH18 chitinase C and *Cycas revoluta* GH18 chitinase A	(GlcNAc)_2_-oxa (GlcNAc)_3_-oxa (GlcNAc)_4_-oxa (GlcNAc)_5_-oxa	(GlcNAc)_5_ (GlcNAc)_4_ (GlcNAc)_3_ (GlcNAc)_2_	(GlcNAc)_7_	[85]
Site-specific chitin dectylases from fungi	(GlcNAc)_4_		A full lineup of partially *N*-acetylated chitotetraoses	[86]

## Data Availability

Not applicable.

## Abbreviations

COS, chitooligosaccharide; DP, degree of polymerization; DA, degree of *N*-acetylation; MW, molecular weight; GlcNAc (**A**), 2-acetamido-2-deoxy-D-glucopyranose; GlcN (**D**), 2-amino-2-deoxy-D-glucopyranose; (GlcNAc)_n_ and (GlcN)_n_, homo-oligosaccharides composed of GlcNAc and GlcN, respectively, with a DP of n; POD, peroxidase; PAL, phenylalanine ammonia lyase; LysM, lysin motif; CERK1, chitin elicitor receptor kinase 1; CERK1-ECD, CERK1 ectodomain; *At*ChiC, a GH18 chitinase from *Arabidopsis thaliana*; *Nt*ChiV, a GH18 chitinase from *Nicotiana tabacum*; *Cr*ChiA, a GH18 chitinase from *Cycas revoluta*; *Bc*ChiA, a GH19 chitinase from *Bryum coronatum*; RSC-c, a GH19 chitinase from *Secale cereale*; TG, transglycosylation.
